# Comprehensive Mining and Characterization of CRISPR-Cas Systems in *Bifidobacterium*

**DOI:** 10.3390/microorganisms8050720

**Published:** 2020-05-12

**Authors:** Meichen Pan, Matthew A. Nethery, Claudio Hidalgo-Cantabrana, Rodolphe Barrangou

**Affiliations:** 1 Department of Food, Bioprocessing and Nutrition Sciences, North Carolina State University, Raleigh, NC 27695, USA; mpan6@ncsu.edu (M.P.); manether@ncsu.edu (M.A.N.); 2 Genomic Sciences Graduate Program, North Carolina State University, Raleigh, NC 27695, USA

**Keywords:** *Bifidobacterium*, CRISPR-Cas, genomics

## Abstract

The clustered regularly interspaced short palindromic repeats (CRISPR)-Cas (CRISPR-associated *cas*) systems constitute the adaptive immune system in prokaryotes, which provides resistance against bacteriophages and invasive genetic elements. The landscape of applications in bacteria and eukaryotes relies on a few Cas effector proteins that have been characterized in detail. However, there is a lack of comprehensive studies on naturally occurring CRISPR-Cas systems in beneficial bacteria, such as human gut commensal *Bifidobacterium* species. In this study, we mined 954 publicly available *Bifidobacterium* genomes and identified CRIPSR-Cas systems in 57% of these strains. A total of five CRISPR-Cas subtypes were identified as follows: Type I-E, I-C, I-G, II-A, and II-C. Among the subtypes, Type I-C was the most abundant (23%). We further characterized the CRISPR RNA (crRNA), tracrRNA, and PAM sequences to provide a molecular basis for the development of new genome editing tools for a variety of applications. Moreover, we investigated the evolutionary history of certain *Bifidobacterium* strains through visualization of acquired spacer sequences and demonstrated how these hypervariable CRISPR regions can be used as genotyping markers. This extensive characterization will enable the repurposing of endogenous CRISPR-Cas systems in *Bifidobacteria* for genome engineering, transcriptional regulation, genotyping, and screening of rare variants.

## 1. Introduction

Clustered regularly interspaced short palindromic repeats (CRISPR) and accompanying CRISPR-associated (*cas*) genes constitute the adaptive immune system in bacteria, which provides resistance against bacteriophage predation [1]. This immunity is orchestrated in three stages. During the first stage, adaptation, snippets of foreign DNA are copied and incorporated into bacterial genomic CRISPR arrays. Next, during the expression stage, the CRISPR array is transcribed and processed to generate mature CRISPR RNA (crRNA) [2,3]. During the last stage, interference, the crRNA guides Cas nuclease(s) for selective target recognition of complementary invasive nucleic acids and subsequent cleavage [4]. Due to the rapid increase in sequencing data and subsequent rise in CRISPR-Cas diversity, the classification of CRISPR-Cas systems is constantly evolving [5]. To date, two classes, six types, and 33 subtypes of CRISPR-Cas systems have been reported. With thousands of CRISPR-Cas systems occurring in nature across genera and species, only a handful have been characterized in detail and repurposed for various applications, notably genetic engineering and transcriptional regulation, among others. Compared to the exponential expansion of CRISPR-Cas applications in eukaryotes, the tremendous application potential in prokaryotes has yet to be fully exploited, particularly in key species related to human health and in food microorganisms. Noteworthy, many human commensal bacteria, probiotic strains, and other industrial workhorses harbor CRISPR-Cas systems in their genomes, allowing the repurposing of these systems for diverse applications without the need of heterologous expression [6]. However, the lack of a fundamental understanding by the scientific community of CRISPR-Cas biology in general, along with the repurposing of endogenous systems in particular, has represented a bottleneck which limits broad implementation.

Bifidobacteria are among the most abundant natural inhabitants of the human gastrointestinal tract, particularly in the infant gut [7,8]. The compositions of infant gut microbiomes differ significantly depending on the delivery and feeding methods, consisting of *Enterobacteriaceae* (around 30%) *Bifidobacterium* (around 10%), some *Lactobacillus* (around 3%), and other diverse bacteria [9]. Their presence is strongly associated with multiple health-promoting effects, although the exact modes of action are yet to be fully revealed. It has been demonstrated that bifidobacteria can modulate the host immune response [10,11], reduce ulcerative colitis and irritable bowel syndrome [12], and ferment non-digestible complex carbohydrates to produce beneficial short chain fatty acids such as butyrate [13]. Due to the potential health benefits, some strains of selected *Bifidobacterium* species have been commercialized as probiotic products [12] which are defined as “live microorganisms that, when administered in adequate amounts, confer health benefits on the host” [14]. Extensive research efforts are underway to study the genomics of bifidobacteria, aiming to discover the underlying mechanisms of their potential health benefits, as well as the genetic relatedness among strains isolated from different hosts and environments [15]. Recent advances in high-throughput sequencing technologies have greatly expanded the availability of bifidobacterial genomes, along with other functional omics data such as transcriptomes and proteomes. These studies have provided insights into the abundance of carbohydrate metabolism systems, adaptations to the glycan-rich gut environment [16], and the diversity of restriction/modification systems [17]. The increase of metagenomic data, together with a new generation of bioinformatic tools to identify and characterize CRISPR-Cas systems [18], has recently allowed for a better understanding of these systems and a wider range of identification across datasets.

CRISPR-Cas based technologies have been gradually implemented for genome engineering in Gram-positive bacteria that are recalcitrant to traditional genetic modification, including *Clostridium* species [19,20], *Lactococcus lactis* [21], and several species of *Lactobacillus* [6,22,23]. Despite the abundance of CRISPR in bifidobacteria, there is a paucity of reports investigating and developing CRISPR applications in bifidobacteria [24,25] and currently no reports on CRISPR-Cas based genome engineering in bifidobacteria.

In this study, we investigated 954 publicly available *Bifidobacterium* genomes to provide insights into the occurrence and diversity of CRISPR-Cas systems in bifidobacteria, identifying all CRISPR-Cas subtypes present across this diverse genus. Then, we performed a characterization of the CRISPR-Cas locus architecture of each subtype and elucidated the essential features required for functional activity and possible development as a genome editing tool, including the prediction of protospacer adjacent motif (PAM), CRISPR RNA (crRNA), and transactivating crRNA (tracrRNA). We hope this work will shed light on the importance and relevance of CRISPR in bifidobacteria and provide a basis for the development of a new generation of genome editing tools.

## 2. Materials and Methods

### 2.1. CRISPR-Cas Systems Detection and Classification

A total of 954 *Bifidobacterium* genomes, omitting repetitive strains, were obtained from the NCBI RefSeq database [26] as of March 2020. CRISPR-Cas identification in each genome was performed using custom Bash and Python pipelines that first identified CRISPR loci using CRISPRViz [27], followed by the extraction of 20 kb flanking regions upstream and downstream of each locus. Using BLAST [28], putative coding sequences in flanking regions were compared against a reference *cas* database assembled from previous reports [29,30,31]. For genomes without identifiable flanking *cas* sequences, all available coding sequences were searched. These results were converted into a Postgres database for subsequent analysis. CRISPR type and subtype classifications were performed according to the identities of signature Cas proteins and other associated genes based on previous reports. The R package “pheatmap” [32] was used to depict the heatmap with the occurrence of CRISPR-Cas systems.

### 2.2. Phylogenetic Analyses

The amino acid sequence alignments of Cas1, Cas3 and Cas9 were performed using the MUSCLE alignment algorithm. Neighbor-joining consensus trees based on the Jukes–Cantor model and 500 bootstrap replications were assembled in Geneious Prime 2020.1 software [33] and the final tree was depicted using FigTree v1.4.4 (http://tree.bio.ed.ac.uk/software/figtree/), without displaying bootstrap values for clarification.

### 2.3. Characterization of CRISPR-Cas Systems Analyses

CRISPR spacers were analyzed and visualized using the CRISPRViz pipeline [27]. The protospacer sequences (corresponding target of the CRISPR spacers) were identified through BLASTn searches of the NCBI nt database. BLAST hits with an e-value smaller than 1e-3 and an identity score greater than 85.0% were included for downstream analyses. Using CRISPRUtils [34], 10 bp upstream and downstream of the identified protospacer were extracted and aligned by CRISPR subtype and species. Then, the predicted PAM sequence was illustrated using the WegLogo server (https://weblogo.berkeley.edu/logo.cgi) [35] based on the conservation of nucleotides at each location. The tracrRNA sequences were identified using BLASTn as previously described [36]. The CRISPR RNA (crRNA) folding structure for Type I CRISPR-Cas systems, and the interaction of the duplex crRNA:tracrRNA were predicted using NUPACK (http://nupack.org/) [37] and depicted by hand.

## 3. Results

### 3.1. Occurrence and Diversity of CRISPR-Cas Systems in Bifidobacterium Genomes

**Figure 1:**
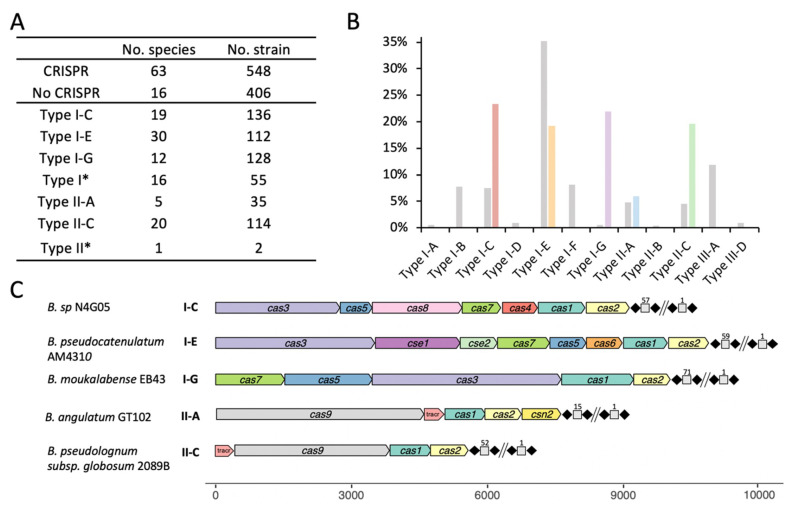
Overall occurrence and diversity of clustered regularly interspaced short palindromic repeats (CRISPR)-Cas (CRISPR-associated *cas*) systems in the genus *Bifidobacterium*. (**A**) The number of species and strains containing CRISPR-Cas systems and each system subtype. Type I* and Type II* represents untyped groups; (**B**) Comparison of CRISPR-Cas occurrence and diversity among *Bifidobacterium* (annotated in color) as compared with all of RefSeq (as of September 2018) annotated in grey; (**C**) One representative CRISPR-Cas locus for each subtype was depicted to demonstrate the locus architecture of *cas* genes and CRISPR array. The long repeat-spacer arrays were shortened for the sake of simplicity and numbered to show the size of the array.

The dramatic advances in high-throughput sequencing technologies have revolutionized the study of genomics, expanding our knowledge of the genus *Bifidobacterium* through continued discovery of novel species. For these analyses, 954 publicly available *Bifidobacterium* genomes from RefSeq, as of March 2020, which encompassed a total of 79 species were used. Overall, 57% (548/954) of bifidobacterial genomes encoded CRISPR-Cas systems (Figure 1A), and displayed a slightly higher occurrence than the 46% prevalence observed across all bacteria [38]. Type I systems (74%) were more prevalent than Type II systems (26%) in *Bifidobacterium* genomes, whereas Type III seemed to be completely absent, based on this dataset. The system, type, and subtype were determined based on the presence of signature Cas proteins (Cas3—Type I, Cas9—Type II) and associated *cas* genes, along with the presence of the CRISPR array. We found 136 strains which contained a Type I-C system, 112 strains which contained a Type I-E system, 128 strains which contained a Type I-G (previously classified as I-U) system (Figure 1A,B). Although Type II systems were less common, 114 strains (19 species) contained Type II-C systems and 35 strains (4 species) contained Type II-A systems. We also reported 55 Type I strains and two Type II strains whose subtype could not be determined based on canonical signature *cas* genes.

The CRISPR-Cas locus architecture, depicted by subtype and illustrating less common bifidobacterial species (Figure 1C), displayed the same (or similar) canonical architecture as previously described [5], containing all the necessary features for a complete and potentially functional CRISPR-Cas system. The Type I systems displayed canonical structure, containing all *cas* genes involved in Cascade (CRISPR associated complex for antiviral defense) complex formation and those required for proper interaction with the crRNA transcript during effector complex formation. Subtypes I-C and I-E followed the canonical layout for their respective subtypes, however, the I-G system of *B. moukalabense* EB43 did not have the *cas8u2* gene and the *cas3* gene was positioned between the *cas5/6* gene and *cas1/4* gene. The signature *cas9* gene of the Type II-A system in *B. angulatum* (4152bp) is slightly larger than the *cas9* of the Type II-C system in *B. pseudolongum* subsp. *globosum* (3429bp). Another unique gene associated with the Type II-A system is the *csn2* gene, located immediately downstream of *cas2*. Type II systems also displayed a tracrRNA required to interact with the repeat sequence of the crRNA to create the duplex crRNA:tracrRNA that guides Cas9 to the targeted nucleic acid.

**Figure 2:**
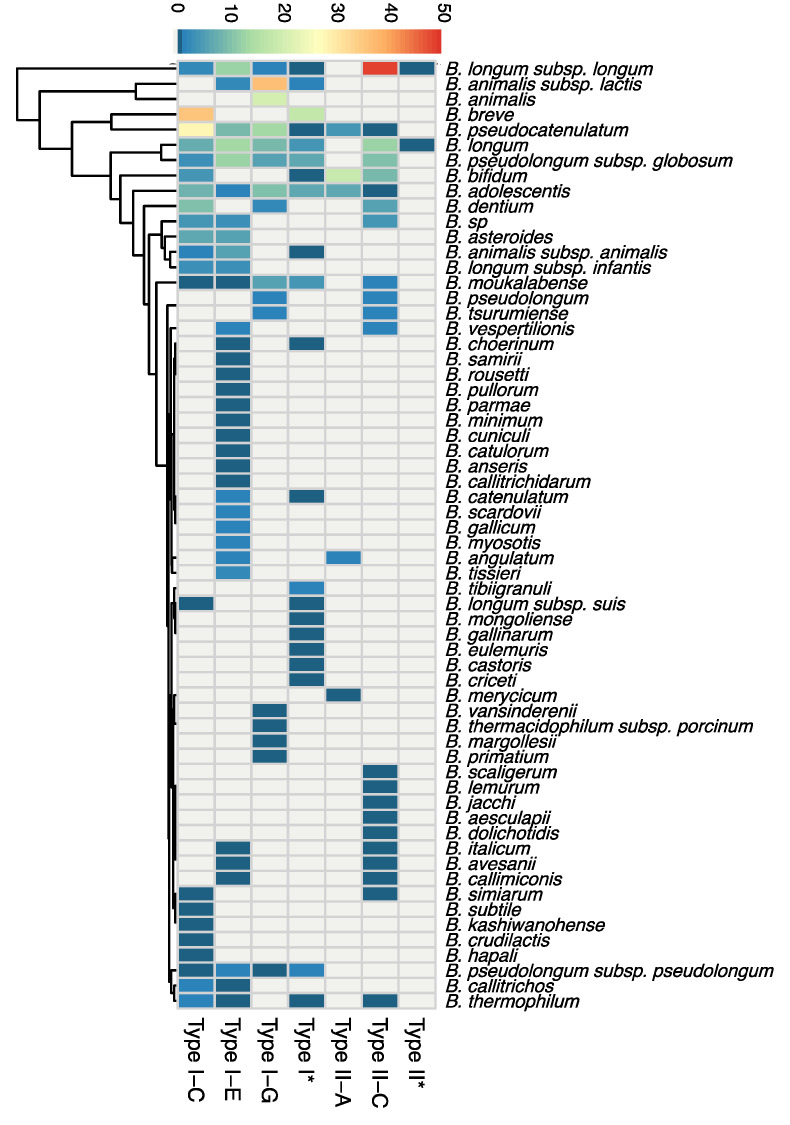
The distribution of 548 CRISPR-Cas systems across species. The heatmap displays the overall number and diversity of CRISPR-Cas system at the species level. Type I* and Type II* represents untyped groups.

The CRISPR-Cas distribution of the 548 detected systems (Figure 2) was highly biased by the number of genomes available for each particular species and subspecies. For newly identified species such as *B. samirii* and *B. tissieri*, there are less publicly available genomes than for well-known species such as *B. adolescentis*, *B. animalis*, or *B. longum*. Overall, Type I-C was present in 16 species, Type I-E in 25 species, and Type I-G in 10 species (Figure 2). Type II-A systems were detected in only five species, whereas Type II-C was more widely distributed, being present in 19 species. As shown in Figure 2, well-studied species such as *B. pseudocatenulatum* and *B. longum* were enriched with diverse CRISPR-Cas systems, with one species covering four distinct CRISPR-Cas subtypes. On the contrary, it seems that the majority of species represented here contain a single distinct subtype (Figure 2). A higher diversity of CRISPR-Cas systems could be revealed in these species over time as the number of available genomes continues to increase.

Distinct CRISPR-Cas systems can exist among strains of the same species and subspecies, indicating that the occurrence of CRISPR-Cas systems is strain dependent and not a general characteristic of the entire species and subspecies. Moreover, some strains can harbor more than one specific CRISPR-Cas system. *B. vespertillionis* RST8 and *B. vespertillionis* RST16 both contain a Type I-E and Type II-C system, with distinct repeat sequences and signature Cas3 and Cas9 proteins. *B. tsurumiense* DSM_17777 and *B. tsurumiense* BSM380WT2B both contain a Type I-G system and a Type II-C system. Notably, when two different CRISPR-Cas systems coexist in the same genome, they belong to different CRISPR types rather than different subtypes, and in some occasions only one locus remains complete and predictively active while the other has missing elements. Despite the strain level characteristics, certain CRISPR-Cas subtypes occur more often than others in selected species. For example, Type II systems do not occur in *B. animalis* subsp. *animalis* or *B. animalis* subsp. *lactis*, whereas several Type I and Type II systems are found in *B. longum* (Figure 2).

### 3.2. Phylogenetic Analyses

**Figure 3:**
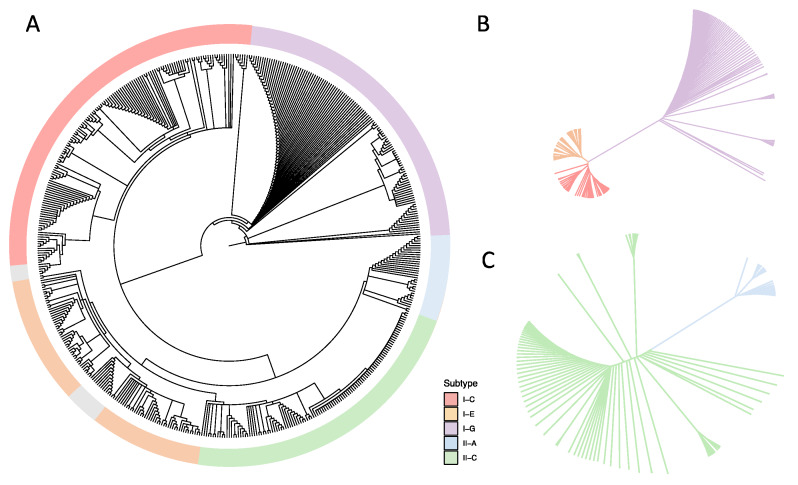
Phylogenetic analyses based on Cas proteins in *Bifidobacterium*. Amino acid sequences of (**A**) Cas1; (**B**) Cas3; and (**C**) Cas9 were aligned using the MUSCLE alignment algorithm. Undetermined CRISPR-Cas subtypes are annotated in grey.

*Cas1* and *cas2* are core genes, involved in spacer acquisition [39], and are present in every CRISPR-Cas system subtype, with their presence being a good indicator of the potential functionality of the CRISPR-Cas system. We performed a phylogenetic analysis based on the amino acid sequence of the Cas1 protein to elucidate the relationship among the identified CRISPR-Cas subtypes and the bifidobacterial species included in this study (Figure 3). Indeed, the Cas1 phylogenetic tree revealed five major branches corresponding to each CRISPR-Cas subtype, independent of the bifidobacterial species. Within the branches representing each CRISPR subtype, the strains of the same species tend to cluster together, revealing that the five main groups are driven by Cas1 sequence identity and that the following subgroups are determined by the species-level identity. Notably, the strains with undetermined CRISPR-Cas subtypes tended to cluster together (Figure 3, gray color). The Cas1 protein in the Type I-G system is a Cas1-Cas4 fusion protein, whose sequence differs significantly from regular Cas1 protein in other subtypes. As a result, the Type I-G system appeared to be its own separate branch away from other systems. Interestingly, the sequence of the Cas1/4 protein was so diverse among *Bifidobacterium* genomes that the Type I-G branch further diverged into multiple subgroups based on species. The Cas1/4 protein in *B. tsurumiense* was the most distinct as compared with other Cas1/4 proteins, with a Blosum62 (threshold 0) score as low as 62%. Likewise, we observed a highly diverse Cas1 amino acid sequence in Type I-E and Type I-C systems. The same was true for Type II-A and Type II-C systems, with the latter being more prevalent in bifidobacterial genomes. The amino acid sequences of the signature Cas3 and Cas9 proteins were extracted to assemble independent phylogenetic trees representing Type I and Type II systems, respectively (Figure 3B,C and Figure S1). This analysis demonstrated that significant variation exists even within the same CRISPR-Cas subtype due to differences in the amino acid sequence in each bifidobacterial species.

### 3.3. Characterization of CRISPR-Cas Systems

**Figure 4:**
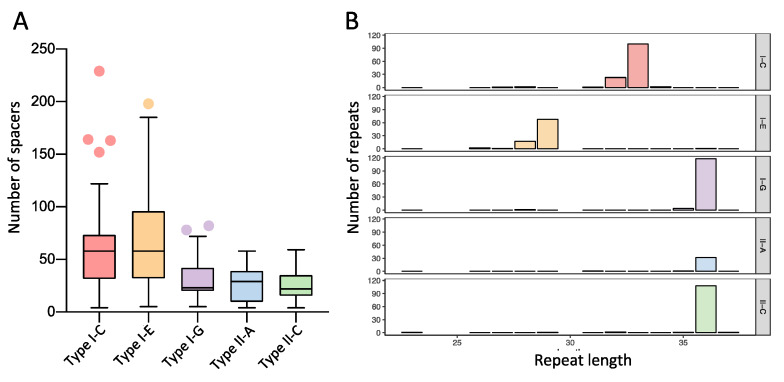
Characterization of CRISPR repeat-spacer arrays in *Bifidobacterium*. (**A**) Distribution of repeat-spacer array size per locus for each CRISPR-Cas subtype; (**B**) Distribution of the repeat length, in nucleotides, for each CRISPR-Cas subtype.

The length of the CRISPR array increases with the number of spacers acquired, with each acquisition event adding one repeat-spacer pair into the preexisting array. Therefore, each CRISPR array always contains “n” repeats and “n *−* 1” spacers. Interestingly, the Type I-C and I-E systems detected here contain longer CRISPR arrays than the Type II systems, averaging 60 spacers in Type I systems and 25 in Type II systems (Figure 4A). The average CRISPR array size for the Type I system in *Bifidobacterium* is longer than the previously reported 40 spacers for a Type I system [40], reflecting the unusually large Type I CRISPR array in *Bifidobacterium*. Type I-E systems displayed the highest average number of spacers (66), representing the majority of longest arrays detected in *Bifidobacterium* genomes, although Type I-C was close behind with an average of 55 repeats. Nonetheless, the distribution of Type I-C and I-E repeat-spacer arrays is highly variable, with as many as 198 spacers in *B. samirii* 2033B and as few as five spacers in *B. longum* AGR2137 for Type I-E CRISPR-Cas systems; and variation between four spacers in *B. pseudocatenulatum* OM108 to 229 spacers in *B. asteroides* W8102 for Type I-C. The length of the Type I-G CRISPR array is similar to that of the Type II systems with an average of 30 spacers, but ranges from 82 spacers in *B. adolescentis* AM1311 to five spacers in *B. dentium* JCVIHMP022. Type II-A and Type II-C systems had a similar average number of spacers, i.e., 26 and 25, respectively. They ranged from as high as 58 for Type II-A or 59 for Type II-C to as low as four spacers for both systems.

Noticeably, the repeat length is usually conserved within the CRISPR subtype, independent of the bifidobacterial subspecies (Figure 4B). We observed the majority of the subtype I-C repeats had 33 nucleotides, whereas the majority of subtype I-E had 29 nucleotides. Interestingly, the vast majority of the repeats in subtype I-G, II-A, and II-C were 36 nucleotides long. This reiterates the differences in subtype I-G as compared with other Type I systems.

**Figure 5:**
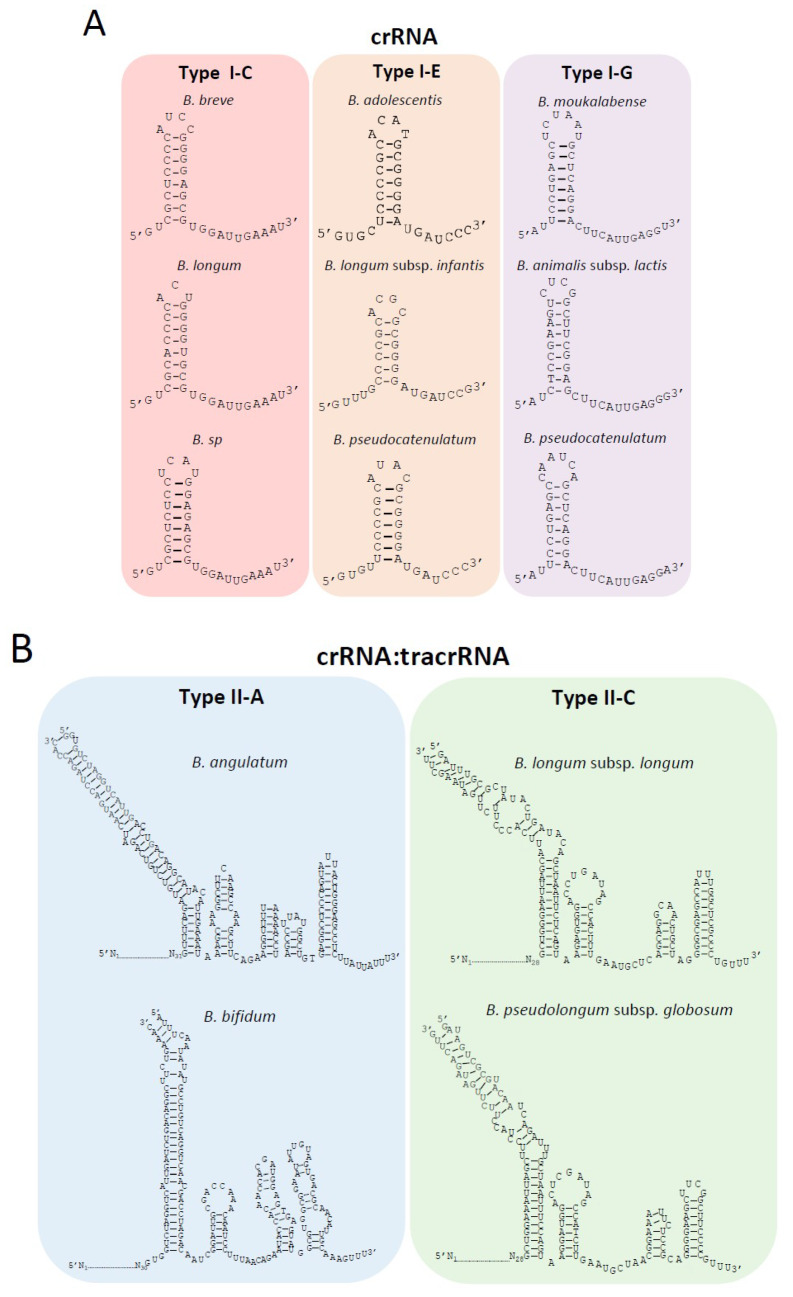
Characterization of crRNA and crRNA:tracrRNA duplex for each CRISPR-Cas subtype in *Bifidobacterium*. (**A**) The conserved crRNA folding hairpin structure is illustrated for multiple species with Type I CRISPR-Cas systems, despite differences in the repeat sequences among different species; (**B**) The crRNA:tracrRNA duplex for Type II systems. The essential elements of the crRNA:tracrRNA duplex are depicted for Type II systems in *Bifidobacterium*, including the upper stem, lower stem, bulge, nexus, and hairpins.

Despite the species-independent conservation of repeat length by subtype, the repeat sequence composition is not well-conserved within each subtype due to sequence variation at the species level (Figure 5). The repeat sequence constitutes the defining feature of an actual CRISPR, and the conserved portion of the CRISPR RNA (crRNA) for both Type I and Type II systems. In Type I systems, the crRNA interacts with the Cascade complex to guide it to a target sequence. Thus, the nucleotide composition of the repeat has a tremendous impact on its secondary structure and affinity for binding Cascade.

Noteworthy, despite the differences in the nucleotide sequence of the repeats between several bifidobacteria species for the same CRISPR-Cas subtype, the final structure of the crRNA is similar for each subtype, with eight nucleotides involved in the base pairing of the hairpin in subtype I-C, 6–7 nt in subtype I-E, and 8–9 nt in subtype I-G (Figure 5A). These hairpin structures lead to the generation of an 11 nt handle at the 3′-end in subtype I-C, a 7–8 nt handle in subtype I-E, and a 12 nt handle in subtype I-G. These 3′-handles are fundamental for the proper function of the CRISPR-Cas system, as it will be processed by Cas5 or Cas6 (subtype dependent), cutting after the seventh or eighth nucleotide from the 3′-end to generate the final mature crRNA.

In Type II systems which lack a Cascade complex and rather rely on a single effector protein, Cas9, the crRNA interacts with another RNA, termed the transactivating RNA (tracrRNA) which is typically present within or adjacent to the CRISPR-Cas locus. The tracrRNAs were identified based on their complementarity to the repeat sequence. The tracrRNA length in subtype II-C was 109 nt for *B. longum* and 108 nt for *B. pseudolongum* subsp. *globosum* (Figure 5B), both of which were located upstream of *cas9* (Figure 1C). The tracrRNA in subtype II-A was 132 nt for *B. angulatum* and 137 nt for *B. bifidum* (Figure 5B), both were located between the *cas9* and *cas1* sequences (Figure 1C). The interaction between both RNAs generates the crRNA:tracrRNA duplex that binds Cas9 and guides it to its nucleic acid target. The interaction between crRNA:tracrRNA was predicted based on sequence complementarity between the tracrRNA and the repeat sequence of the crRNA (Figure 5B). The dual crRNA:tracrRNA architecture for both Type II subtypes displayed a canonical layout, including the lower and upper stem of the bulge, the nexus, and the terminal hairpin structures. Proper folding of the crRNA:tracrRNA duplex, particularly at the bulge and nexus, is essential for efficient Cas9 endonuclease activity and is typically conserved. Within subtype II-C, the structure of the nexus and two terminal hairpins were conserved, while the II-A subtype presented a different nexus structure and three terminal hairpins (Figure 5B).

### 3.4. Spacer Homology Search and PAM Prediction

**Figure 6:**
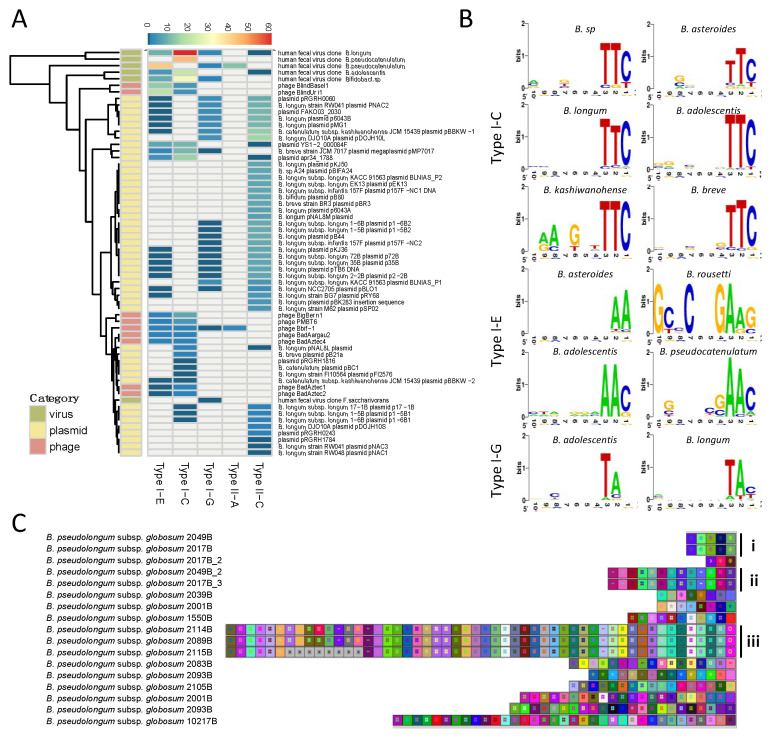
Spacer characterization and homology search. (**A**) Spacer homology search was conducted through BLASTn and hits were categorized into three categories: virus, plasmid, and phage. The number of hits per CRISPR-Cas subtype was determined and depicted; (**B**) The protospacer adjacent motifs (PAMs) were predicted using protospacer flanking sequences and were illustrated with WebLogo. Nucleotide height represents the conservation of that nucleotide at that specific location; (**C**) The spacers in *B. pseudolongum* subsp. *globosum* were extracted and aligned using CRISPRViz.

Spacers represent the hypervariable region of the CRISPR array, and each spacer sequence corresponds to a segment of DNA procured from an invading phage or invasive plasmid. The source nucleic acid of the spacer sequence is named the protospacer [41]. Matching the spacer to its protospacer via BLAST allowed us to identify the origin of several spacer sequences. Overall, the majority of positive hits matched bacteriophages and plasmids (Figure 6A). Importantly, not every spacer queried yielded a matching protospacer, representing a bottleneck in this analysis. For example, a total of 80 spacers from *B. angulatum* strains were searched and only two positive hits were detected. In another case, there was a total absence of spacer-protospacer homology for over three hundred spacers in *B. pseudolongum* subsp. *globosum*.

The protospacer adjacent motif (PAM), a short nucleotide sequence (3–5 nt) adjacent to the protospacer sequence, represents another essential element for effective nucleic acid target recognition [42,43]. The PAM sequence is commonly located at the 5′-end of the protospacer in Type I systems and at the 3′-end in Type II systems. The PAM was determined for the Type I-C, I-E, and I-G subtypes, however, the PAM sequence for subtypes II-A and II-C was not clear. Interestingly, the PAM sequence of each Type I subtype was conserved across species, namely for subtype I-C which displayed a highly conserved PAM 5′-TTC-3′ (Figure 6B, right panel). For subtype I-E, the PAM has some variability among species with several potential PAMs such as 5′-AAC-3′ or 5′-GAAG-3′. Subtype I-G displayed a PAM of 5′-TAN-3′.

The robustness of PAM prediction depends on the number and quality of positive hits obtained from spacer-protospacer homology searches, with a higher confidence on the predicted PAM with more positive hits with high levels of sequence homology. In this regard, the homology search for Type I-C in *Bifidobacterium sp* was performed on 317 spacers, resulting in 12 positive hits. These protospacers belonged to uncultured human fecal virus, *Mycobacterium* phage, and plasmid apr34_1788. The 5′ flanking region of the protospacers was well-conserved, displaying a 5′-TTC-3′ PAM sequence for Type I-C. Similarly, 15 positive hits were generated from 672 spacers in *B. pseudocatenulatum*, belonging to uncultured human fecal virus and *Bifidobacterium* phage PMBT6. The conserved 5′ flanking region of the protospacer displayed a 5′-AAC-3′ PAM sequence for Type I-E in *B. pseudocatenulatum*. The predicted PAM for Type I-G was 5′-TAC-3′ in *B. moukalabense* based on two positive hits from homology search against 318 spacers. Although this prediction can be inconclusive due to limited hits, it is in concordance with PAMs previously predicted for Type I-G (previously I-U) in *B. longum*, 5′-TAT-3′ [24].

Finally, CRISPR spacers can be used to decipher strain evolution based on the similarity of the spacer sequence across strains, the number of spacers, and the order of acquisition, reflecting historical vaccination events. This has been extensively used for genotyping of bacterial pathogens and to a lesser extent, starter cultures and probiotic strains. Here, we show strain genotyping for the species *B. pseudocatenulatum* subsp. *globosum* using its subtype II-C spacers (Figure 6C). Strains of distinct phylogenetic origin contain different ancestral spacers (spacers on the right) and generate different subgroups over time, with divergent spacer content as illustrated in subgroups i-iii. Within subgroups i and ii, the spacer content of the strains seemed to be identical. In subgroup iii, the three strains were almost identical except that the strain 2115B lost eight recently acquired spacers.

## 4. Discussion

**Figure 7:**
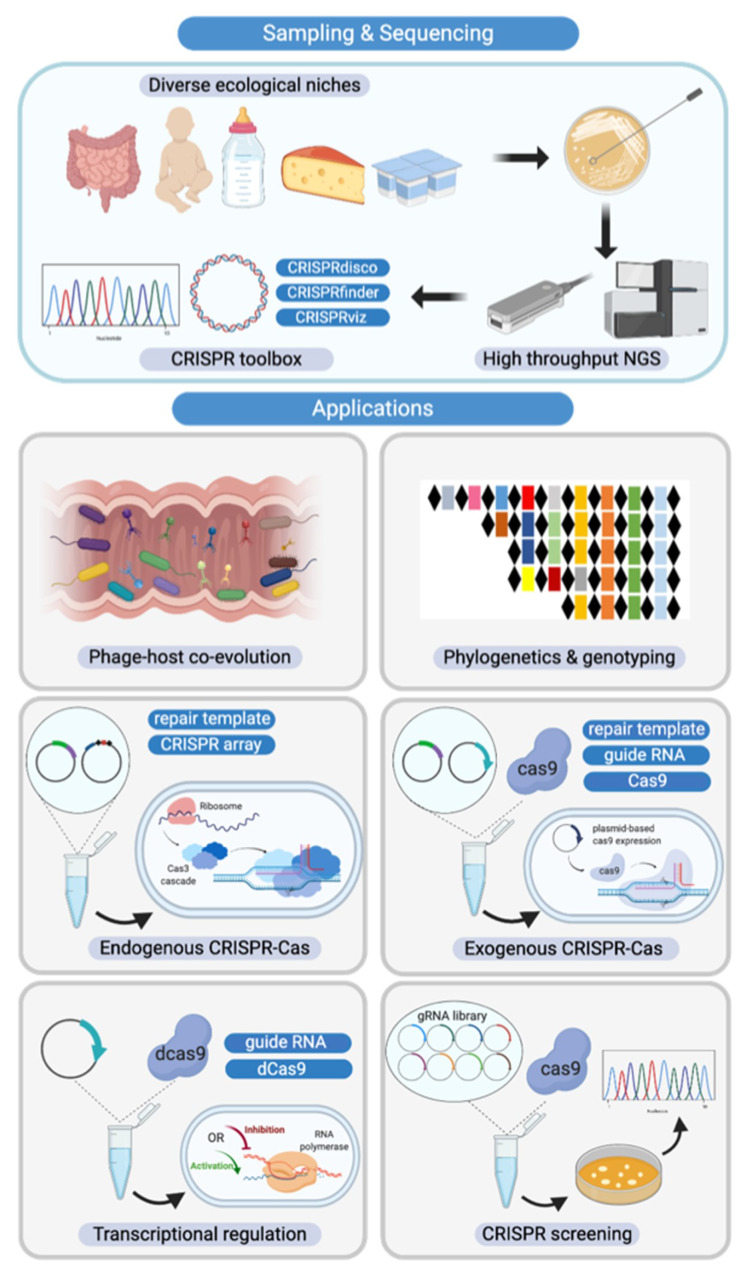
Diverse CRISPR-Cas system applications in *Bifidobacterium*. (**Top Panel**) Novel *Bifidobacterium* species and CRISPR-Cas systems discovered through high-throughput next generation sequencing, assisted by a plethora of bioinformatic analysis tools; (**Bottom Panel**) From left to right and then top to bottom. CRISPR-Cas systems serve as the bacterial adaptive immune system, influencing the predator-prey dynamics between phages and commensals such as *Bifidobacterium* in the human gastrointestinal tract. CRISPR arrays can also be used as unique genetic markers for strain identification and genotyping. As discussed in this study, CRISPR-Cas systems are highly abundant in bifidobacterial genomes. With in-depth analysis of CRISPR elements such as crRNA, tracrRNA, and PAM reported here, we have built the platform for repurposing these endogenous CRISPR-Cas systems as genome editing tools. In the case of absence of CRISPR-Cas systems or lack of functionality, exogenous CRISPR-Cas systems can be delivered using plasmid-based systems. Beyond genome editing, alternative CRISPR-Cas systems with deactivated nucleases can serve as transcriptional regulation tools. In light of next generation sequencing (NGS), guide RNA libraries can be prepared to screen natural *Bifidobacterium* variants in a high-throughput fashion, bypassing the genetic modification route that can require strict regulation.

As the cost of next generation sequencing (NGS) has significantly decreased in the last decade, genome availability has expanded drastically. Over 300 *Bifidobacterium* genomes were deposited into RefSeq in 2019 alone. Thanks to NGS, we now have a diverse collection of *Bifidobacterium* genomes, revealing previously unknown species and broad genomic diversity [15,44] (Figure 7). Concurrently, a plethora of bioinformatic tools such as CRISPRdisco [38], CRISPRfinder [45], and CRISPRViz [27] have been developed to mine and characterize novel CRISPR-Cas systems. The advances in high-throughput NGS, along with continued development of bioinformatic tools, have set the stage for continued discovery and characterization of CRISPR-Cas systems with a variety of applications.

Genomes of bifidobacteria showcase a myriad of CRISPR-Cas systems, providing extraordinary potential for repurposing these naturally occurring systems for various applications, ranging from deciphering phage-host coevolution, to strain genotyping and development of next generation genome editing tools. To date, there are only three reports on the characterization of CRISPR-Cas systems in *Bifidobacterium*, all with limited datasets [24,25,46]. Here, we used a substantially larger dataset including all publicly available *Bifidobacterium* genomes in RefSeq, encompassing 79 species, 954 strains, and five CRISPR subtypes. While the CRISPR-Cas subtypes identified were the same as those previously reported [25], Briner et al. did not detect CRISPR-Cas systems in *B. longum* subsp. *longum* and *B. longum* subsp. *infantis* and did not subtype the CRISPR-Cas system in *B. longum* subsp. *suis*; whereas Hidalgo-Cantabrana et al. described subtypes I-C, I-E, I-G, and II-C across all three *B. longum* subspecies [24], which was consistent with our findings. These data demonstrate the presence of CRISPR-Cas systems in *Bifidobacterium* is strain dependent, rather than species dependent, making estimations of the overall occurrence quite challenging. It is likely that as more novel bifidobacterial species are sequenced, more CRISPR-Cas systems will be detected, expanding the described occurrence of each subtype.

Spacer homology searches revealed limited matches to plasmids, phage, and prophage sequences, highlighting one of the main hurdles in characterizing and applying CRISPR-Cas systems in bacteria. Compared to the exponential growth in the availability of bacterial genomes, there is a relative paucity of publicly available phage sequences. The fact that hundreds of spacers are of unknown origin suggests that many bacteriophages or plasmids are yet to be discovered or sequenced. Still, the hypervariable CRISPR array provides a unique opportunity for genotyping. On the basis of the spacer alignment in our study, several strains shared the same ancestral spacers at the beginning of the array and gradually diverged and acquired different spacers, evolving into different strains under various selective pressures, including pressure from various invasive genetic elements. The use of CRISPR spacers for genotyping has been previously demonstrated in *Streptococcus thermophilus* [42] and other food microorganisms, representing a powerful tool for strain identification and traceability [47].

The extensive characterization of CRISPR-Cas systems in the human commensal *Bifidobacterium* performed in this analysis allowed us to elucidate all the essential elements for repurposing these endogenous systems for various applications, including genome editing and transcriptional regulation. Although transformation protocols using *Escherichia coli*-*Bifidobacterium* shuttle vectors have been established, such systems work only in limited species with low transformation efficiency [48]. The complex cell wall structure along with restriction and modification systems has made bifidobacteria notoriously recalcitrant to genome editing. Repurposing the diverse endogenous CRISPR-Cas systems in *Bifidobacterium* for genome editing purposes holds tremendous potential (Figure 7), as this has been done in many other bacteria [6,49]. Taking advantage of endogenous CRISPR-Cas systems avoids transforming plasmids containing large *cas* sequences. To achieve targeted mutagenesis, a short CRISPR array and repair templates are transformed into the cell for targeted cleavage and subsequent repair via homologous recombination [6]. In the case where no native system exists or the endogenous system is not functional, the heterologous expression of CRISPR elements can be delivered through a plasmid-based system. In order to survive in the human gastrointestinal tract, bifidobacteria and other commensal bacteria have evolved to metabolize host-derived glycans such as human milk oligosaccharides (HMOs) and other glycoproteins and glycolipids present at mucosal surfaces [50,51]. Endogenous or exogenous CRISPR-Cas systems can be used to create bifidobacteria mutants to study glycan utilization pathways and identify novel key enzymes and metabolic regulators.

As aforementioned, Bifidobacteria can ferment a variety of human- and plant-derived glycans and their associated metabolites are thought to confer a range of health benefits upon their hosts, particularly infants [52]. To date, there is limited knowledge regarding the transcriptional control of the pathways that enable bifidobacteria to be among the early colonizers of the human gut. Using CRISPR-based transcriptional regulation systems, such as CRISPR interference (CRISPRi), could reveal more pathway checkpoints for regulation while discovering new ways to modulate the production of desired metabolites [53]. CRISPRi systems can be constructed either through delivery of a deactivated Cas9 protein on a plasmid, deactivating an endogenous *cas9* naturally existing in the chromosome, or through deletion of the active nuclease protein (such as Cas3 protein in Type I-E system) to achieve transcriptional repression [54]. An endogenous Type I-E CRISPR-Cas system was repurposed to regulate metabolic flux in *E. coli*, redirecting the majority of the flux away from the central metabolic pathway to poly-3-hydroxbutyrate (PHB biosynthesis pathway) [55]. CRISPR-Cas systems can also be repurposed to screen for rare natural variants in bifidobacteria, bypassing the strict regulation on genetically modified organisms when selecting for probiotic strains with desirable but rare phenotypes (Figure 7) [56,57]. Natural mutants that either have a mutated sequence or deletion will escape crRNA targeting while the wild-type strains will be killed through lethal DNA cleavage [57]. Considering its recalcitrance to genome editing and the strict GMO regulation, this could be a promising strategy to screen for natural variant strains in *Bifidobacterium.*

In this study, we presented a comprehensive screening of CRISPR-Cas systems in all publicly available *Bifidobacterium* genomes in the NCBI RefSeq database. We observed diverse CRISPR-Cas systems spanning five different subtypes, with large and distinct CRISPR loci containing a myriad of spacers that provided insights into bifidobacteria strain evolution and predator–prey dynamics. We further characterized the essential elements such as crRNA, tracrRNA, and PAM sequences for all five CRISPR subtypes in different species. This work lays the foundation for repurposing CRISPR-Cas systems in bifidobacteria for a variety of applications ranging from genome editing and transcriptional control, to rare variant screening and genotyping. Altogether, we envision the wide utilization of CRISPR-Cas systems to expedite the development and formulation of next generation *Bifidobacterium* probiotics.

## Supplementary Materials

The following are available online at https://www.mdpi.com/2076-2607/8/5/720/s1.
