# Supplementary material for: Comprehensive Mining and Characterization of CRISPR-Cas Systems in Bifidobacterium
Source: Microorganisms. 2020 May 12;8(5):720. doi: 10.3390/microorganisms8050720 (PMC7284854; doi:10.3390/microorganisms8050720)
Supplement: Supplementary file 1 [file microorganisms-08-00720-s001.pdf]

microorganisms

MDPI

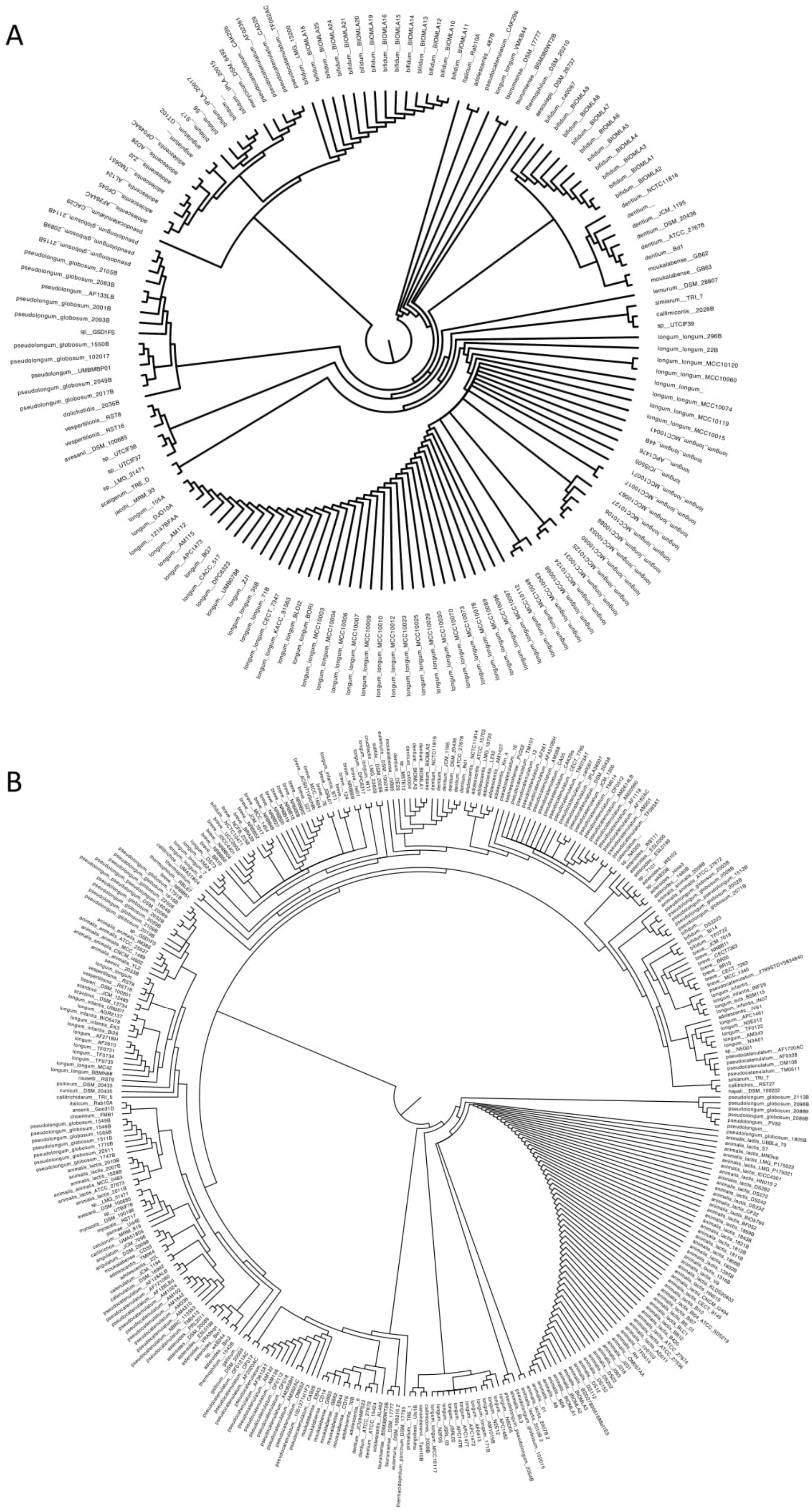

Figure S1: Phylogenetic analyses based on the amino acid sequences of (**A**) Cas 9 protein and (**B**) Cas3 protein in Bifidobacterium, with taxa names shown. The amino acid sequences were aligned using the MUSCLE alignment algorithm to generate neighbor-joining consensus trees based on the Jukes-Cantor model.
